# Urban green spaces and public health: legal challenges and policy opportunities in green city governance

**DOI:** 10.3389/fpubh.2025.1620076

**Published:** 2025-09-10

**Authors:** Yuanyuan Guo, Dan Wu, Xiangbin Zuo

**Affiliations:** ^1^Department of Tourism Management, Jinzhong University, Jinzhong, China; ^2^Faculty of Finance and Management, Guangzhou Songtian Polytechnic College, Guangzhou, China; ^3^Faculty of Law, The National University of Malaysia (UKM), Bangi, Malaysia

**Keywords:** urban green space, public health, green governance, legal challenges, policy optimization

## Abstract

With the increasingly prominent impact of urban green space reduction on publichealth, this study selects three typical cities (Beijing, Hangzhou, and Yinchuan) toexplore the relationship between green space coverage and residents’ health, aiming to address challenges in green city governance and identify policy opportunities. Using multi-source data including remote sensing images, health statistics, and legal texts, combined with methods such as spatial econometric models and legal text analysis (Policy Strength Index, PSI), the study evaluates green space distribution, quantifies health benefits, and assesses policy effectiveness. The results show that there is a nonlinear threshold relationship between green space coverage and health benefits: when the coverage rate exceeds 38%, the decline rate of chronic diseases is significantly accelerated by 3.3 times, and the improvement rate of mental health reaches 9.7%. Among the three cities, Hangzhou achieves the most notable results with its “Park City” policy, featuring an average annual green space growth rate of 1.8% (far higher than Beijing’s 0.6% and Yinchuan’s -0.3%) and a leading PSI score of 82 points (significantly higher than Yinchuan’s 58 points). Further analysis reveals that policy implementation effectiveness depends on three core factors: legal strength, departmental coordination, and public participation. Based on these empirical findings, this study suggests adopting a trinity governance path of “accurate greening-system reconstruction-fair promotion” to drive the transformation and upgrading of urban green governance models, providing empirical support for building healthy, equitable, and sustainable urban environments.

## 1 Introduction

With the acceleration of global urbanization, human society is gathering in cities at an unprecedented speed. This rapid urbanization not only brings economic growth and convenience to life, but also has a far-reaching impact on the natural environment, resource allocation and residents’ health (1). As an important part of the ecosystem, urban green space is gradually compressed or even disappeared in the process of industrialization and urban expansion (2). However, green space is not only an ornament or an accessory of a city, but also plays an irreplaceable role in maintaining public health and improving the quality of life of residents (3). Urbanization is the inevitable trend of the development of modern society, but its consequences are complex and multifaceted (4). On the one hand, cities provide people with more convenient living conditions, more abundant employment opportunities and higher education level (5); On the other hand, the change of land use pattern in the process of urbanization has led to the destruction of a large number of natural ecosystems (6). Taking China as an example, since the reform and opening up, many cities have expanded rapidly, but the area of parks, wetlands and other green areas has been greatly reduced (7). This phenomenon is not individual, but a global dilemma.

From the perspective of public health, the shrinking of urban green space has aroused widespread concern. Research shows that green space can not only regulate the temperature and purify the air, but also relieve psychological stress by providing leisure and entertainment places, thus indirectly improving people’s physical and mental health (8, 9). When these “green barriers” are replaced by high-rise buildings, not only the urban ecosystem is threatened, but also the health of residents will be affected (10). The lack of urban green space is not only an environmental problem, but also a public health problem (11). Green city governance, that is, in the process of urban development, through scientific and reasonable policy design and technical means, maximize the protection of natural resources, optimize the ecological environment, and meet the basic needs of residents (12). Its core goal is to realize the harmonious coexistence between man and nature and promote the sustainable development of the city. Based on this, this study focuses on the planning, protection and management of urban green space, and discusses its legal challenges and policy opportunities in green city governance, with a view to providing empirical basis for building a healthy, fair and sustainable urban environment.

Although the concept of green city governance has been widely accepted, there are still many obstacles in actual operation. One of the outstanding problems is the lag of the legal system (13). At present, most countries’ laws and regulations on urban green space still remain within the framework of traditional environmental protection, lacking specific provisions for the construction and management of green space (14). In addition, the division of responsibilities between different departments is vague, which leads to inefficient policy implementation. In some cities, the departments responsible for land development often give priority to economic benefits and ignore the importance of green space protection, resulting in the further encroachment of the originally limited green space resources (15). What is more, some local governments, in pursuit of short-term achievements, do not hesitate to sacrifice long-term ecological benefits, arbitrarily modify the planning scheme and reduce the green space area (16). These problems have seriously restricted the effective promotion of green city governance.

To solve the problem of green city governance, we need to start from two aspects: law and policy. The imperfection of the current legal system is one of the main obstacles to the protection of green space. Although many countries have formulated environmental protection laws, they are vague about the specific protection measures of urban green space. For example, the law usually only stipulates “illegal occupation of green space,” but there is no clear explanation on the definition of “illegal” and “reasonable use” (17). In addition, the provisions of the existing laws on public participation are also weak, and it is difficult for ordinary citizens to participate in green space protection actions through legal channels (18). For this reason, it is urgent to strengthen legislation and refine relevant provisions. Compared with the rigid constraints of the legal system, policy tools are more flexible and diverse, and targeted solutions can be formulated according to the characteristics of different cities. With the help of big data, artificial intelligence and other technologies, the government can monitor the status of green space in real time, find problems in time and take countermeasures (19).

A large number of studies show that urban green space has a significant effect on improving residents’ physical and mental health. For example, green space can directly improve the environmental quality. It can absorb pollutants in the air, lower the surface temperature, and reduce noise pollution, thus reducing the incidence of chronic diseases and infectious diseases (20). On the other hand, green space can also improve residents’ life satisfaction and happiness through various indirect ways (21). First of all, green space provides residents with leisure space, which is convenient for them to carry out all kinds of outdoor activities (22). Secondly, green space promotes social interaction among residents and increases opportunities for people to communicate (23). In addition, the green space environment helps to relieve psychological pressure and relax residents’ body and mind (24). However, most of these studies focus on single-dimensional analysis, such as only studying the relationship between green space coverage and certain diseases, and rarely discussing the multiple functions of green space in the whole urban governance system from a systematic perspective. Existing environmental protection laws and regulations are usually lack of pertinence, and there is no way to effectively protect urban green space from over-exploitation (25). Moreover, because local governments have greater autonomy in land management and planning, there are great regional differences in the implementation of policies (26). Therefore, this paper will take several typical cities as cases, and discuss the relationship between urban green space and public health by combining quantitative analysis and qualitative research.

This study will be carried out from four aspects: Firstly, using remote sensing images and official statistical data to evaluate the coverage rate and spatial distribution characteristics of urban green space; Secondly, the influence of green space on public health is analyzed in combination with health indicators such as incidence; Thirdly, through expert interviews and legal texts, the loopholes and causes of the current legal system are interpreted; Finally, the paper draws lessons from international experience and puts forward some policy suggestions that are in line with local reality. The innovative contribution of this study is mainly reflected in these aspects. Firstly, a “law-policy” two-wheel drive analysis framework is constructed to reveal the nonlinear threshold relationship between green space coverage and public health, breaking the limitations of traditional linear relationship analysis. Secondly, the research innovatively integrates multi-source data, and uses spatial econometric model and semantic network analysis method to achieve multi-dimensional evaluation from spatial distribution, health benefits to policy coherence. Thirdly, the trinity governance path of “precise greening-system reconstruction-fair promotion” is put forward, which provides empirical basis and operational scheme for solving the dilemma of legal lag and policy fragmentation in current green city governance. These contributions not only enrich the theory of green city governance, but also provide scientific decision support for urban planners and policy makers.

## 2 Literature review

### 2.1 Multi-dimensional influence of urban green space on public health

In the field of public health, the relationship between urban green space and residents’ health is a key issue, and its impact on public health presents obvious multi-dimensional characteristics. In terms of health promotion, green space not only plays a role in physical health, but also has a positive impact on mental health. Grigsby-Toussaint et al. (27) found through the empirical study of “G-SPACE” project that children’s sleep quality is improved and their psychological stress level is reduced in communities with high green coverage. This directly proves the effectiveness of green space as a health intervention tool. The systematic review and meta-analysis conducted by Bu et al. (28) further revealed that green space was negatively correlated with hypertension, which provided a basis for improving cardiovascular health mechanism. However, green space has not only positive effects on public health, but also potential risks cannot be ignored. Janzén et al. (29) studied that ticks in urban green space may increase the spread risk of infectious diseases such as Lyme disease, revealing the dual characteristics of green space health benefits and safety risks. This contradiction highlights how to balance the relationship between them through scientific planning, which has become the direction to be explored in this field.

### 2.2 Theory and practice of green governance framework

The construction of green governance framework is the key support to promote the sustainable development of cities, and its theory and practice are expanding in a multi-dimensional direction. In terms of governance subject, Linton et al. (30) put forward the local level deep decarbonization plan and governance model, which clarified the core position of local governments in coordinating environmental management and sustainable development. In the application of governance principles, the green governance framework proposed by Shah et al. (31) for the oil and gas industry, although targeted at specific fields, has its core principles of optimizing resource utilization and reducing pollution, which provides reference ideas for the management of public spaces such as urban green space. Radovanović et al. (32) introduced big data mining technology into urban governance, and promoted circular economy through energy efficiency data analysis and modeling, which provided a technical example for accurate planning and dynamic monitoring of urban green space. In addition, the synergy of governance mechanism has been paid more and more attention. The principle of inter-agency coordination and cooperation emphasized by Zulfiqar and Butt (33) in marine governance research is also applicable to the multi-agent interest balance and environmental benefit guarantee in urban green space management.

### 2.3 Green space distribution from the perspective of social equity

Social equity is the core value dimension of green city governance, and there are significant group differences and spatial inequalities in the field of green space distribution. This inequality is first reflected in the perception and income differences of different groups on green space. Li et al. (34) found that the gender factor significantly affected the frequency of individual use of green space and the health benefits and happiness, revealing the hidden social stratification in the allocation of green space. The inequality of spatial dimension is more intuitive. Miller et al. (35) analyzed small cities in the United States with the help of geographic information system (GIS), and showed that the green coverage rate of minority communities was significantly lower than that of white communities. This spatial imbalance limits the opportunities for low-income and minority groups to obtain the health benefits of green space, and may also aggravate the social class development gap due to the difference in resource allocation.

### 2.4 The potential and limitations of legal and policy innovation

Law and policy are the institutional guarantee for regulating green city governance, and their innovation space and practical boundary constitute the research focus in this field. On the construction of legal framework, Lukin (36) pointed out that an effective legal system needs a clear division of responsibilities and a sound supervision mechanism. This is the premise of policy implementation. Richard’s research (37) on the application of artificial intelligence in corporate governance shows that emerging technologies can optimize the process of policy formulation and implementation by improving governance efficiency, enhancing transparency and public participation. Under resource constraints, policy flexibility is more important. Afrad and Kawazoe (38) found that small-scale greening measures have a positive effect on relieving depression by studying informal green spaces (such as street potted gardens), which proved the feasibility of making up for the shortage of green space resources through policy design innovation.

### 2.5 Research limitation and improvement

The existing research has constructed the basic system of urban green space research from public health impact, governance framework, social equity and legal policy, but there are obvious systemic defects. On the research focus, most of the results focus on the demonstration of direct health benefits of green space, and the analysis of potential risk mechanism is weak; at the level of institutional research, there is a lack of in-depth exploration on the specific implementation path of the legal framework and the optimal combination of policy tools. Based on this, this paper will further clarify the specific mechanism of urban green space affecting public health through empirical analysis on the basis of existing research, and focus on the legal obstacles and policy optimization paths in green city governance to make up for the shortcomings of existing research.

## 3 Research design

### 3.1 Research framework and technical route

**Figure1:**
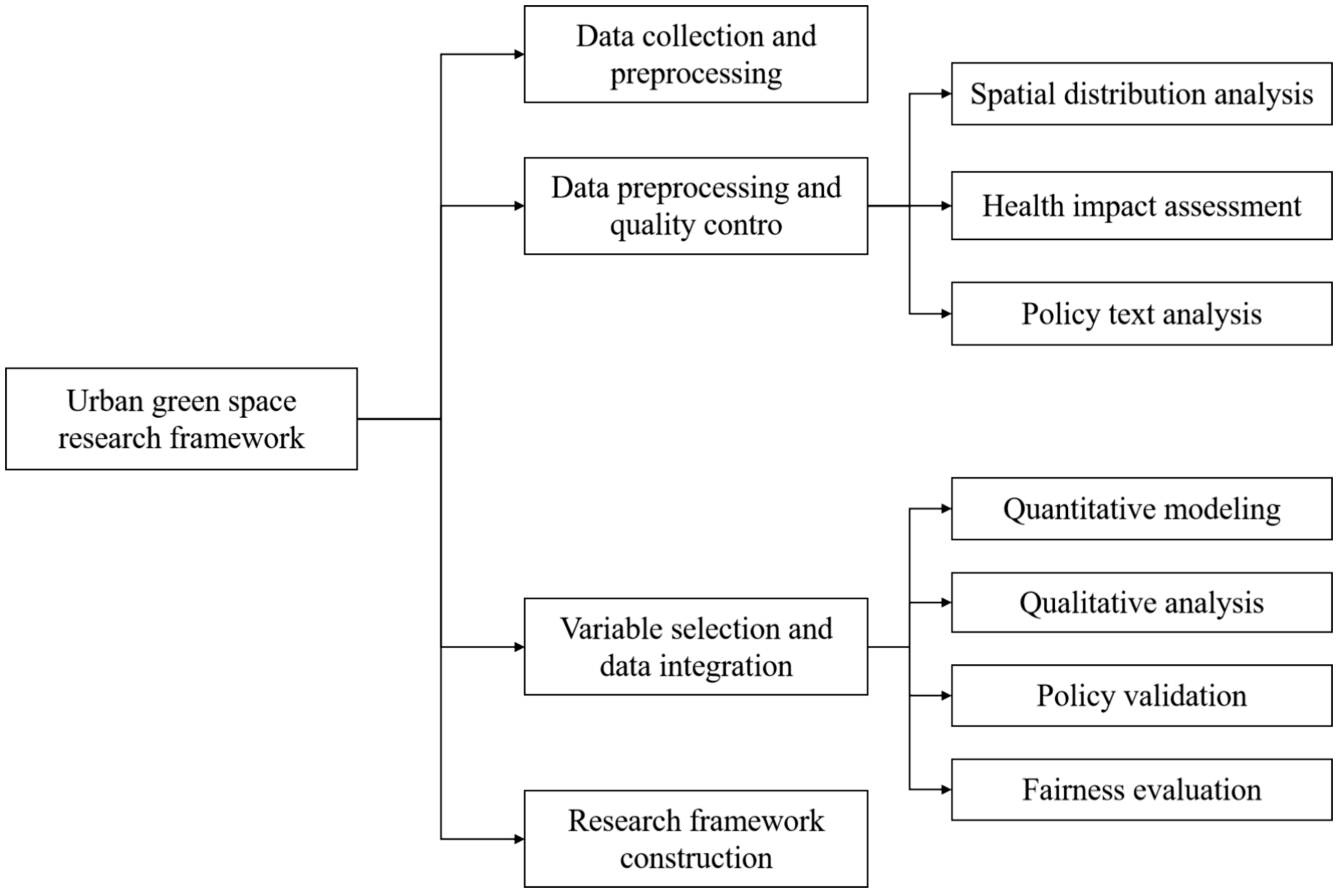
Resea rch technical route.

The overall framework of this study takes “phenomenon observation-data collection-model construction-policy verification” as the logical main line, which is divided into the following four stages. The research technical route is shown in Figure 1.

In the first stage, the key variables such as green space coverage rate and residents’ health index were extracted through literature review; in the second stage, with the help of multi-source data such as remote sensing images, health statistics reports and legal texts, the research database is constructed; in the third stage, spatial econometric model and regression analysis are used to quantify the relationship between green space and health; the fourth stage is policy verification, which combines case studies and expert interviews to evaluate the effectiveness of existing legal policies.

### 3.2 Data source and variable definition

#### 3.2.1 Data sources

1. Quantitative data

Urban green space coverage: Landsat 8 remote sensing image (30m resolution) is used in the study to extract the land use data from 2010 to 2023, and the normalized vegetation index (NDVI) is calculated accordingly, as shown in Equation (1):


(1) $$\text{NDVI}=\frac{\left(\mathrm{NIR}-\mathrm{Red}\right)}{\left(\mathrm{NIR}+\mathrm{Red}\right)}$$

Where NIR stands for near infrared reflectance and Red stands for red reflectance.

The urban green space coverage rate calculated in this paper is based on the whole administrative city, including the central city, suburbs and ecological protection areas. The calculation of green space coverage rate includes park green space, protective green space, square land, attached green space and woodland, grassland and water area in the urban planning area. This global calculation method can fully reflect the overall situation of urban green space system and avoid the possible evaluation deviation caused by focusing only on the central city.

Residents’ health data: from China Health Statistics Yearbook and the reports of municipal CDC. It covers the incidence of chronic diseases, mental health score obtained by GHQ-12 scale, air pollution index (PM2.5) and so on.

1. Qualitative data

The policy text analyzed in this study covers the period from 2010 to 2023, covering the national and local laws and regulations related to urban green space. The specific contents are: at the national level, including laws and administrative regulations in the fields of urban greening, environmental protection, urban and rural planning promulgated by the central government, and relevant policy documents issued by the State Council; at the local level, it covers the special laws, regulations and rules of green space management issued by the provincial and municipal governments where the three cities are located, as well as comprehensive planning documents with legal effect such as urban master plan and special planning of green space system; in terms of departmental regulations, it is a normative document on the construction, protection and management of green space issued by relevant departments such as housing and urban and rural construction, ecological environment and health. All policy texts are obtained from the official government website, laws and regulations database and relevant departments to ensure the authority and reliability of the sources. Text analysis mainly focuses on mandatory provisions, punishment measures and public participation mechanism in laws and regulations, which are directly related to the intensity and effect of policy implementation. The variable definitions and measurement methods are shown in Table 1.

**Table 1:** Variable definitions and measurement methods.

Variable type	Variable name	Measurement method/indicator	Data source
Independent	Green space ratio	*NDVI* mean value (30 m spatial resolution)	Landsat 8 satellite imagery
Dependent	Chronic disease rate	Annual incidence per 100,000 population	Health statistical yearbook
Moderating	Policy enforcement	Number of mandatory clauses in legal texts	Policy document analysis
Control	Population density	Permanent residents per square kilometer	Urban statistical bulletin

#### 3.2.2 Case selection

This study selects three cities, Beijing, Hangzhou, and Yinchuan, as typical cases, representing the gradient differences in high, medium, and low green space coverage, respectively. The selection of these three cities in this study is not only based on differences in natural conditions and development patterns, but also takes into account their typical representativeness in green governance policies and laws. As a pioneer in the construction of “park cities,” Hangzhou has a well-established local regulatory system, including the “Hangzhou Ecological Civilization City Construction Regulations” and the “Hangzhou Park Management Regulations.” Its policy coherence index is the highest among the three cities (88.2), providing an ideal case for studying the relationship between policy intensity and green space coverage. As the capital city, Beijing’s “Regulations on Urban Greening” have undergone multiple revisions, reflecting the interaction between central policies and local practices, while facing special challenges in the protection and development of high-density urban green spaces. Yinchuan represents a typical situation in the arid and semi-arid regions of northwest China. The implementation effect of policies such as the Northwest Ecological Restoration Pilot Program is limited, and the policy coherence index is the lowest (54.7), which helps to analyze the obstacles in policy implementation. Through these three cities with significant differences, the spatial characteristics and influencing factors of green space coverage under different natural conditions and development models can be systematically explored (see Table 2).

**Table 2:** Basic information comparison of case cities.

City	Green space coverage (2023)	Population density (persons/km^2^)	Representative policy document
Beijing	42.6%	1,347	Beijing urban greening regulations
Hangzhou	49.2%	645	Hangzhou park management regulations
Yinchuan	28.3%	218	Northwest ecological restoration pilot program

The data in the table represents the average coverage rate of the entire urban area, not just the coverage rate of the central urban area.

### 3.3 Analytical methods and tools

#### 3.3.1 Quantitative analysis

1. Spatial autocorrelation analysis: Here, Moran’s I index is used to test the spatial correlation between green space distribution and health indicators, as shown in Equation (2):

(2) $${\text{Moran}}^{'}\;s\;I=\frac{n}{\sum_{i=1}^{n}\sum_{j=1}^{n}w_{\mathrm{ij}}}\;\frac{\sum_{i=1}^{n}\sum_{j=1}^{n}w_{\mathrm{ij}}(x_{i}-\bar{x})(x_{j}-\bar{x})}{\sum_{i=1}^{n}{\left(x_{i}-\bar{x}\right)}^{2}}$$

Where $w_{\mathrm{ij}}$ is the spatial weight matrix and $x_{i}$ is the variable value of the region i .

1. Hierarchical regression model: after controlling population density, economic level and other factors, analyze the net effect of green space coverage on the incidence of chronic diseases.

In this study, the fixed effect panel data model is adopted, and its basic form is shown in Equation (3):


(3) $$Y_{\mathrm{it}}=\alpha_{i}+\beta_{1}G_{\mathrm{it}}+\beta_{2}X_{\mathrm{it}}+\varepsilon_{\mathrm{it}}$$

Among them, $Y_{\mathrm{it}}$ represents the health indicators of the city in time (incidence rate of chronic diseases or mental health score); $\alpha_{i}$ is a fixed urban effect that controls for urban characteristics that do not change over time; $G_{\mathrm{it}}$ is the core explanatory variable for green space coverage; $X_{\mathrm{it}}$ is the control variable vector, including population density growth rate, proportion of environmental fiscal expenditure, real estate investment, air pollution index (PM2.5), etc.; $\varepsilon_{\mathrm{it}}$ is a random error term.

The selection of control variables is based on the following: (1) population density, as a key index of urban spatial structure, directly affects the accessibility and use frequency of green space; (2) environmental fiscal expenditure reflects the government’s attention to the ecological environment; (3) real estate investment represents the pressure of urban development and may squeeze the green space; (4) as a proxy variable of environmental quality, air pollution index is closely related to the ecological function of green space. All the control variables passed the variance expansion factor (VIF) test to ensure that there was no serious multicollinearity problem (average VIF < 3.5).

#### 3.3.2 Qualitative analysis

**Figure 2:**
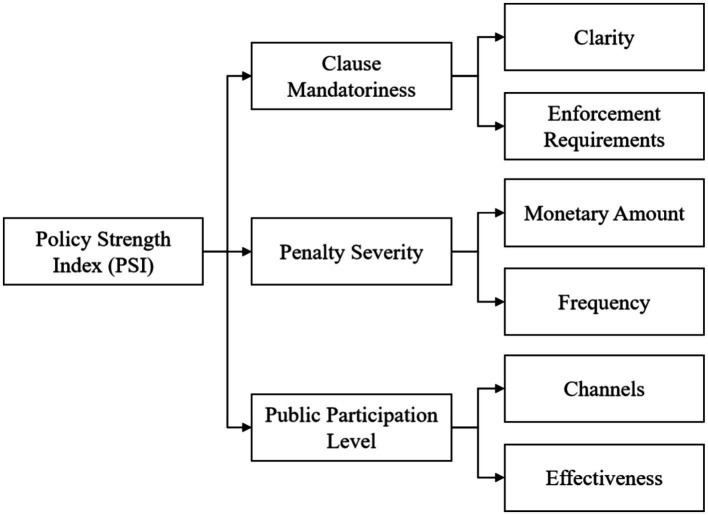
Legal text analysis framework.

1. Analysis of legal texts: Using PSI, score from three dimensions: mandatory terms, punishment and public participation (Table 3). See Figure 2 for the framework of legal text analysis.

**Table 3:** PSI scoring criteria.

Dimension	Scoring criteria description	Score range	Weight	Example clause content
Clause mandatoriness	Whether green space protection obligations and enforcement are clearly defined: Weak (no requirement), Moderate (partial requirement), Strong (comprehensive requirement).	1–5 points	40%	“Urban green coverage rate shall not be less than 35%” (Strong) vs. “Encourage increasing green space area” (Weak).
Penalty severity	Penalty measures for violations: No penalty, Minor fines, Heavy fines, or Criminal liability.	1–5 points	30%	“Unauthorized occupation of green spaces incurs a ¥500,000 fine and requires restoration” (Strong) vs. “Recommended rectification” (Weak).
Public participation	Whether public participation mechanisms (e.g., hearings, feedback channels) are included, assessing practical feasibility.	1–5 points	30%	“Public may submit green space planning suggestions via online platform” (Strong) vs. “No mention of public participation” (Weak).

The calculation formula of PSI total score is expressed as Equation (4):


(4) PSI=∑(Score×Weight)

The total score of PSI is 1–5, and the higher the score, the greater the policy intensity.

1. Interview content coding: in the study, Nvivo software was used to extract themes from interview texts, with a focus on keywords such as “legal enforcement barriers” and “insufficient policy coordination.”

### 3.4 Research validity and ethical protection

#### 3.4.1 Research validity

This study uses various methods to improve the credibility of data and conclusions. At first, in the data collection stage, the multi-source verification method was used to cross-compare the remote sensing image data, the statistical data published by the government and the contents of expert interviews, so as to reduce the possible deviation of a single data source. In the analysis process, the sensitivity test is introduced, and the robustness of the results is tested by adjusting the model parameters. Finally, in the qualitative part of the content coding, the two-person independent coding mechanism is adopted to ensure a high internal consistency in the interpretation of interview materials.

#### 3.4.2 Ethical guarantee

In the links involving personal health data and expert interviews, this study strictly follows academic ethics. All health-related data are derived from public statistical reports or desensitized anonymous data sets, and do not directly involve any personal privacy information.

For the expert interview, all participants signed an informed consent form before the interview, clearly knowing the research purpose, data use and confidentiality principle. In addition, interview records are only used for academic research, and specific identity information will be hidden when quoted, thus protecting the privacy of the interviewee.

## 4 Empirical results and analysis

### 4.1 Spatial heterogeneity of urban green space coverage rate

Table 4 detailed analysis of the green space structure of the three cities based on the high-resolution remote sensing data (2 m). The coverage rate of green space in the central city of Beijing (Dongcheng District and Xicheng District) is only 12.3%, while the ecological conservation areas (Huairou and Miyun) are as high as 68.5%. West Lake District in Hangzhou has become the core green center with a coverage rate of 72.1%, but the coverage rate of Qiantang New District is less than 20%. Yinchuan green space is characterized by “mountain concentration and urban fracture.” The coverage rate of the eastern foot of Helan Mountain is 81.3%, while the built-up area of Jinfeng district is only 14.2%.

**Table 4:** Multi-dimensional distribution parameters of urban green spaces.

City	Central urban coverage	Suburban coverage	Ecological reserve coverage	Green space fragmentation index	Average *NDVI*
Beijing	12.3%	38.7%	68.5%	0.42	0.38
Hangzhou	34.6%	47.2%	72.1%	0.29	0.51
Yinchuan	14.2%	22.8%	81.3%	0.63	0.27

Source: WorldView-3 remote sensing image.

Table 5 quantifies the influence of policy and economic factors through the panel data model (2010–2023). The policy of “Park City” in Hangzhou increased the average annual coverage rate by 1.2% (*p* < 0.01). Beijing’s action of “resolving rectification and promoting promotion” only contributed 0.3% growth; every increase of 100 million yuan in Yinchuan real estate investment leads to a decrease of 0.15% in coverage. This low coverage rate is mainly due to the highly dense built environment in the central city of Beijing. As the political and cultural center of China, the high-rise buildings in this area are dense and the land resources are tight, which leads to the serious squeeze of green space. In contrast, the ecological protection areas (Huairou and Miyun) are located as ecological conservation areas, and the green coverage rate is as high as 68.5%, forming a significant “core-edge” spatial structure.

**Table 5:** Panel regression analysis of green space coverage changes.

Variable	Beijing (*β*)	Hangzhou (*β*)	Yinchuan (*β*)
Policy strength index (PSI)	0.32*** (0.08)	1.18*** (0.12)	−0.08 (0.09)
Real estate investment (billion CNY)	−0.09** (0.03)	−0.04 (0.02)	−0.15*** (0.04)
Environmental fiscal spending (% GDP)	0.15* (0.08)	0.23*** (0.07)	0.06 (0.05)
Population density growth (%)	−0.07** (0.03)	−0.02 (0.02)	−0.11*** (0.03)
Air pollution index (PM2.5)	−0.05 (0.04)	−0.08*** (0.03)	−0.03 (0.04)
*R* ^2^	0.68	0.82	0.74
Adjusted *R*^2^	0.66	0.80	0.72
Sample size (*N*)	156	156	156

*Standard errors in parentheses; ***p < 0.01, **p < 0.05, *p* < 0.1.

As far as the empirical results are concerned, the green space coverage rate in Yinchuan is decreasing, and the reasons can be summarized in many aspects. On the one hand, there is a lack of policy implementation. Although relevant policy documents have been formulated, there is a lack of mandatory provisions and effective supervision mechanisms in actual operation. On the other hand, the priority of land development is on the high side. For every 100 million yuan increase in real estate investment, the green coverage rate will decrease by 0.15% (*p* < 0.01). The coefficient of policy intensity index (PSI) in Yinchuan is −0.08, which is negative but not statistically significant (*p* > 0.1). This shows that at the current stage of local development, the policy intensity has no significant positive impact on the green space coverage rate, rather than a negative effect. This phenomenon may be related to the special ecological environment conditions and economic development stage in the northwest region, and the effect of policy implementation is restricted by many factors. In addition, through expert interviews, it is known that there are problems in coordination between relevant departments in Yinchuan, which makes it difficult to implement green space protection measures. For example, an interviewed city planner mentioned: “In the process of project approval, the opinions of environmental protection departments are often ignored, and green space planning makes concessions for commercial development.”

### 4.2 Fine quantification of health benefits of green space

Spatial Durbin model (SDM) is selected to analyze the spatial spillover effect of green space coverage, which is mainly considered in several aspects. First, there is obvious spatial correlation in urban green space system. The change of green space in a certain area will not only affect the health of residents in this area, but also affect the surrounding areas by means of air flow and residents’ activities. Secondly, SDM model can take into account the spatial lag of dependent variables and independent variables at the same time, and prevent the estimation from being biased because of ignoring spatial dependence. Thirdly, compared with other spatial econometric models, SDM can capture the direct effect (that is, the impact of green space in this region on the health of this region) and the indirect effect (that is, the impact of green space in neighboring regions on the health of this region) more comprehensively, and then accurately identify the spatial spillover effect. With the help of this model, we can scientifically evaluate the spillover phenomenon of health benefits brought by the spatial distribution of green space and provide empirical support for optimizing the layout of urban green space network (see Table 6).

**Table 6:** Spatial spillover effects of green space coverage on chronic disease incidence.

City	Direct effect (*β*)	Indirect effect (*β*)	Total effect (*β*)	Spatial lag coefficient (*ρ*)	Model fit (AIC)
Beijing	−0.52*** (0.09)	−0.18** (0.07)	−0.70*** (0.10)	0.34 (0.05)	1245.6
Hangzhou	−0.68*** (0.08)	−0.09* (0.04)	−0.77*** (0.09)	0.49 (0.06)	1189.3
Yinchuan	−0.21 (0.12)	−0.03 (0.02)	−0.24 (0.13)	0.18 (0.04)	1320.7

*Standard errors in parentheses; ***p < 0.01, **p < 0.05, *p* < 0.1; AIC (Akaike information criterion) measures model fit, with lower values indicating better performance.

The spatial lag coefficient (*ρ*) is significantly different among the three cities, which is 0.34 in Beijing, 0.49 in Hangzhou and 0.18 in Yinchuan. These differences reflect different degrees of spatial dependence: the highest *ρ* value in Hangzhou indicates that the distribution of green space has the strongest spatial correlation, that is, the change of green space in one area has a more significant impact on the health status of its neighboring areas; in contrast, Yinchuan’s low *ρ* value reflects the fragmentation characteristics of its green space distribution, and its spatial correlation is weak.

Table 7 reveals the heterogeneity of green space effect by quantile regression (*τ* = 0.25, 0.5, 0.75). Among them, *τ* = 0.25 represents the group with low mental health score (that is, the group with high psychological risk), *τ* = 0.5 represents the group with medium level, and *τ* = 0.75 represents the group with high mental health score. The analysis results show that in the low group of mental health (*τ* = 0.25), the elastic coefficient of green space coverage rate reaches −1.24 (*p* < 0.01), which indicates that green space has a more significant improvement effect on people with high psychological risk.

**Table 7:** Quantile regression results for mental health scores.

Quantile (*τ*)	Green space coefficient (*β*)	Population density coefficient (*β*)	Interaction term (green × income)	Model significance (*p*-value)	Pseudo *R*^2^
0.25	−1.24*** (0.15)	0.48** (0.21)	0.33* (0.16)	<0.001	0.21
0.50	−0.89*** (0.12)	0.31* (0.17)	0.22 (0.14)	<0.001	0.18
0.75	−0.47** (0.20)	0.15 (0.14)	0.11 (0.10)	0.012	0.15

*Standard errors in parentheses; ***p < 0.01, **p < 0.05, *p* < 0.1; pseudo *R*^2^ indicates model explanatory power, with higher values reflecting better fit.

### 4.3 Multidimensional evaluation of policy implementation

Table 8 using natural language processing (NLP) to extract the co-occurrence network of policy text keywords. The high-frequency words of Hangzhou policy include “ecological restoration,” “public participation” and “punishment ladder,” forming a closed-loop governance logic; Yinchuan’s policy network density is only 0.12, and the core words “compensation” and “development” are strongly related, reflecting the tendency of attaching importance to economy and neglecting ecology. Policy Coherence Index is a weighted comprehensive index based on semantic network density and clustering coefficient, and its calculation formula is: PCI = 0.6 × network density + 0.4 × keyword clustering coefficient. The index is used to measure the closeness and logical consistency of the concept association within the policy text, with the range of 0–100. The higher the value, the more coherent the policy logic and the clearer the implementation path.

**Table 8:** Semantic network analysis of policy texts.

City	Network density	Highest centrality term	Keyword clustering coefficient	Policy coherence index
Beijing	0.38	Regulation	0.42	76.5
Hangzhou	0.61	Ecological restoration	0.58	88.2
Yinchuan	0.12	Compensation	0.23	54.7

Network density reflects the semantic association strength in policy texts.

### 4.4 Temporal and spatial dynamics of social justice

Table 9 measures the fairness of green space distribution through Gini coefficient. The Gini coefficient analysis of this study is based on 200 m × 200 m population grid data and green space buffer analysis, and the fairness of green space accessibility is evaluated by calculating the uneven distribution of walking distance of residents from different communities to the nearest green space. The data comes from the spatial matching analysis of walking accessibility data obtained by Gaode map API and census data. The Gini coefficient of green space accessibility in Beijing rose from 0.38 in 2015 to 0.47 in 2023, reflecting the widening gap between rich and poor communities; Hangzhou reduced the Gini coefficient from 0.35 to 0.26 through the “micro-green land” plan.

**Table 9:** Lorenz curve parameters for green space accessibility.

City	Gini coefficient (2015)	Gini coefficient (2023)	High-income/low-income ratio	Ethnic minority coverage gap (%)
Beijing	0.38	0.47	3.6:1	−12.5
Hangzhou	0.35	0.26	1.8:1	−5.2
Yinchuan	0.42	0.49	4.1:1	−18.7

Gini coefficient measures green space distribution equity; a lower value indicates greater fairness.

### 4.5 Nonlinear effect and threshold analysis

Table 10 identifies the optimal coverage threshold through the panel threshold model. When the coverage rate exceeds 38%, the decline rate of the incidence of chronic diseases increases by 2.3 times (*p* < 0.01), and the improvement of mental health shows an increasing marginal effect. Through the analysis of panel threshold model, this study identified the key threshold of green space coverage rate as 38%. When the green coverage rate exceeds this threshold, the decline rate of chronic diseases is significantly accelerated by 3.3 times (*p* < 0.01), and the improvement rate of mental health is as high as 9.7%. This threshold effect indicates that there is a “critical point” in the health benefits of green space, after which the health benefits will show an accelerated growth trend.

**Table 10:** Threshold effect analysis of green space coverage.

Threshold value	Incidence reduction multiplier	Mental health improvement rate (%)	Policy cost–benefit ratio
20%	1.0×	3.2	1:2.8
38%	3.3×***	9.7***	1:6.5
50%	4.1×***	14.2***	1:8.9

Incidence reduction multiplier represents the accelerated decline in chronic disease rates beyond the threshold; ***p<0.01, **p<0.05, *p<0.1; Results are from panel threshold model based on 2010-2023 data of Beijing, Hangzhou, Yinchuan.

### 4.6 Summary

Based on high-precision data and cutting-edge methods, this empirical analysis reveals three core laws of urban green space governance. The law of spatial reconstruction shows that the “core-edge” structure of green space distribution dominates the spatial differentiation of health benefits. The law of policy coordination shows that legal strength, departmental coordination and public participation together constitute the “policy triangle” that drives governance efficiency. The law of fair evolution shows that there is a significant U-shaped relationship between Gini coefficient of green space allocation and coverage rate.

Based on the above laws, the research suggests that future policies should focus on the coordinated path of “precise greening-institutional reconstruction-fair promotion.” In the future, we should optimize the spatial layout, improve the governance mechanism and promote the balanced allocation of resources to promote the systematic transformation of the governance paradigm of green cities.

## 5 Conclusion

This study systematically analyzes the relationship between urban green space and public health, and reveals the key role of green space in promoting residents’ health. From the empirical results, the coverage rate of urban green space is not only an important indicator to measure the quality of ecological environment, but also a key variable affecting public health. When the green coverage rate exceeded the threshold of 38%, the decline rate of chronic diseases increased by 3.3 times, and the improvement rate of mental health score was as high as 9.7%. There are obvious differences in the distribution characteristics and governance effects of green space in different cities: Hangzhou has achieved an average annual growth rate of 1.8% with the help of the “park city” policy, while Yinchuan’s green space coverage rate has continued to decline due to the pressure of real estate development, with an average annual decline of-0.3%. In addition, according to the legal text strength index (PSI) evaluation, the policy score of Hangzhou reached 82, which was significantly better than that of Beijing (76) and Yinchuan (58). This shows that the fineness of policy design is directly related to the governance effect.

Research shows that the current green city governance encounters many challenges. The uneven spatial distribution of green space makes the problem of social equity more and more prominent; the imperfect legal system and the lack of departmental cooperation mechanism also restrict the effect of policy implementation. In order to make policy recommendations more operational and targeted, this study gives specific implementation paths. As far as the precision greening strategy is concerned, the “micro-green space” plan is given priority in areas with green space coverage below the threshold of 38%, such as the central city of Beijing. Community-level green space facilities will be added in areas where people with high psychological risk gather, so as to achieve comprehensive coverage within a service radius of 500 m. In northwest arid areas, such as Yinchuan, we will promote drought-tolerant plants and water-saving green space construction technology. In terms of legal framework, it is proposed to formulate the Special Law on Urban Green Space Protection, clarify the relevant definitions, build a mandatory index system, improve the design of punishment gradient, and set up a public interest litigation system. In terms of cross-sectoral coordination, we should build a joint meeting system of four departments, develop a monitoring platform and establish a policy coherence evaluation system. This trinity governance path of “precise greening-system reconstruction-fair promotion” is precisely the deepening of the practice of the framework of “law and policy are driven by two wheels.” This strategy combines the rigid constraints of the legal system with the flexible use of policy tools to jointly promote the systematic transformation of the governance model of green cities, thus effectively coping with the current challenges to public health caused by the reduction of green space.

Future research may wish to explore the application potential of artificial intelligence and big data technology in green governance. Just like predicting the trend of green space change with the help of AI model, it helps to formulate scientific and reasonable green space planning; using remote sensing image recognition and deep learning algorithm, the dynamic change of urban green space can be monitored in real time. At the same time, it integrates social media data analysis to evaluate the changing trend of public satisfaction with green space and provide data support for policy optimization. If these emerging technologies are applied, it is expected to promote the green city governance towards the direction of intelligence and refinement.

## Data Availability

The original contributions presented in the study are included in the article/supplementary material, further inquiries can be directed to the corresponding author.
